# Factors associated with producing a scientific publication during medical training: evidence from a cross-sectional study of 40 medical schools surveyed in Latin America

**DOI:** 10.12688/f1000research.26596.1

**Published:** 2020-11-24

**Authors:** Mario J. Valladares-Garrido, Christian R. Mejia, Annel B. Rojas-Alvarado, Mary M. Araujo-Chumacero, Jhacksson S. Córdova-Agurto, Jessica Fiestas, Feeder J. Rojas-Vilar, Carlos Culquichicón

**Affiliations:** 1 Universidad Continental, Lima, Peru; 2 School of Medicine, Universidad Privada Antenor Orrego, Piura, Peru; 3 CI- Emerge, Center of Emerging Diseases and Climate Change, Universidad Nacional de Piura, Piura, Peru; 4 School of Health Sciences, Universidad Nacional de Piura, Piura, Peru; 5 School of Medicine, Universidad Cesar Vallejo, Piura, Peru; 6 Facultad de Medicina, Universidad Ricardo Palma, Lima, Peru

**Keywords:** Medical Education, Undergraduate, Scientific Societies, Latin America, Medical Students

## Abstract

**Background: **Scientific publication during medical training is key to promote enduring cutting-edge knowledge. The promotion of science among medical students in Latin America is a multi-sphere issue hampered by the unawareness of governments to invest in national research, as well as a lack of support from local universities. This study aims to determine the factors associated with producing a scientific publication during medical training among Latin American medical students of local scientific societies.

**Methods: **This is a secondary data analysis of a cross-sectional study initially conducted in 2016 to evaluate the use of information and communications technologies (TICs) among medical students of 40 local scientific societies of medical students affiliated to the Latin American Federation of Medical Students Scientific Societies (FELSOCEM, in Spanish). Teams in each local scientific society surveyed self-reported scientific publications and explored its association with socioeconomic, academic, and research training conditions. We included medical students enrolled in the 2016-I term and excluded medical interns. We implemented nested models to identify covariates associated with self-reported scientific publication until reaching a parsimonious mixed-effect multilevel model clustered by medical scientific society.

**Results: **We surveyed 11,587 medical students. The prevalence of scientific publications increased in 36% among medical students affiliated to a Scientific Society of Medical Students [parsimonious prevalence ratio (PRp)=1.36, 95%CI=1.16–1.59], 51% among medical students with advanced English proficiency [PRp=1.51, 95%CI=1.21 – 1.87], 85% among medical students who attended a scientific writing skills course [PRp=1.85, 95%CI=1.59–2.15], 81% among medical students who use Sci-Hub [PRp=1.81, 95%CI=1.50–2.20], and 108% among medical students who have access to a pirated academic account [PRp=2.08, 95%CI=1.83–2.36].

**Conclusions: **Producing a scientific publication among medical students is associated with being affiliated to a Scientific Society of Medical Students, English proficiency, training in scientific writing, use of Sci-Hub, and pirated academic accounts.

## Introduction

 Producing a scientific publication during medical training is key to promote the constant medical training and to encourage students to create cutting-edge knowledge; in this way, students will build-up research skills and critical thinking, and conduct evidence-based practice and patient-centered care with an endured vision to follow a scientific career ^1– 3^. Latin American universities are progressively recognizing the critical importance of foster science in the early onset of undergraduate, and are implementing research-oriented courses such as research design methods, biostatistics, epidemiology, and a final research-centered thesis ^4^. However, there are still gaps in Latin America when comparing to research university systems from developed countries in terms of the number of publications, the quality of articles published, the outreach of the studies, and funding opportunities ^5^. Studies in Colombia and Brazil show that medical students consider scientific research as an important issue of their training and the low scientific production is influenced by the lack of inspirational and committed mentors as role models for the beginning of a scientific career ^6, 7^. Between 1997 and 2010, there was an increase of 8.4% in student participation in manuscripts published on journals indexed in Scielo-Peru, of whom 42% reported being affiliated to a scientific society of medical students ^4, 8^.

 In Peru, the progress of research in medical undergraduate has been strongly promoted by the Peruvian Scientific Society of Medical Students (SOCIMEP, by its acronym in Spanish), an organization that has been improving the research training of medical students for 27 years ^9^. SOCIMEP stands scientific and academic committees in their central and 38 local scientific societies throughout local medical schools of Peru, and held international, national, and local scientific conferences ^9^. The SOCIMEP also foster societies to actively participate and integrate them into a nation-wide research network, and provide connections with experienced research mentors. Being affiliated to the SOCIMEP's local scientific society is associated with increased scientific production (PR: 2.41; 95% CI: 1.55-3.74) ^10^. However, only 10% of the projects conducted in local scientific societies are published in indexed journals due to deficient methods implemented in the studies, lack of knowledge about editorial processes, few local mentors and a lack of financial support from public agencies and institutions ^11^. The funding opportunities for medical students are poor in the local medical schools of Peru and in much of Latin America; overall government investment is disproportionately granted to attend local public health priorities with well-implemented laboratories, and full-time research-centered faculties ^12^. In Peru, less than 30% of universities have research funding programs for students to implement their thesis research operations, or research student program awards ^13^.

 The promotion of science among medical students in Latin America is a multi-sphere issue hampered by the unawareness of governments on the investment in nation research and innovation well-structured systems, as well as a lack of local universities support, their lack of investment in research facilities, and a lack of international-research experienced mentors ^3^. In this scenario, our study aims to determine the factors associated with scientific publication during medical training, in order to identify the needs of Latin American local scientific societies for the implementation of their continuing education programs in research. Our hypothesis is that there are factors from medical training associated with producing a scientific publication during undergraduate.

## Methods

### Study design

This is a secondary data analysis of a cross-sectional study initially conducted in 2016 to evaluate the use of information and communications technologies (TICs) in medical students along Latin America ^14, 15^. This study evaluated 40 scientific societies of medical students from Latin America. We primarily evaluated the self-report of scientific publications and used the following variables to explore its associated factors: gender, age, university, current year of career, affiliated to a scientific medical student society, English proficiency, studied previous career, courses in scientific databases including PubMed, Scopus and Scielo, courses in scientific writing skills, courses in scientific browsing, courses in Zotero, use of Sci-Hub, access and provider of pirated academic accounts.

### Population and sampling

The primary study surveyed 11,587 students from 40 medical schools, including two from Ecuador, two from Panama, four from Paraguay, three from Bolivia, 18 from Peru, two from Mexico, two from Venezuela, one from Honduras, three from Colombia, one from Chile, and two from Argentina. This study included medical students enrolled in the term 2016-I and excluded who were doing the internship.

We made a stratified sampling using the academic year in the medical school as a stratum. The estimated sample size for each investigation site was 289 medical students, we also added 10% to anticipate withdraws. Thus, we aimed to survey 318 medical students in each university. We consider a sample size calculation with an 80% of power, and 5% of significance for an infinite sample size. Regarding participant selection, the interviewer team entered the course with greatest credits in each academic year and picked the students who were sat in an odd numbered location per row. In three universities, the sample size was not enough large to reach the minimum required, so we conducted a census-type sampling.

### Operational procedures

In 2015, the ICTs project was awarded on the category of multi-center projects by the XXX International Congress of the Latin American Federation of Medical Students Scientific Societies (FELSOCEM, by its acronym in Spanish). This award let connect the researchers within the FELSOCEM’s international collaboration network and carry out the study. We could enroll teams from 40 out of 69 Scientific Medical Students Society (SOCEMs, by its acronym in Spanish) along Latin American. Each scientific society had at least one team with three medical students who were trained on scientific integrity ^16^, standardized methods for survey participants, data entry procedures, and quality control of datasets.

In each medical school, every team of interviewers surveyed at the beginning or the end of the lectures, prioritizing that the students had long time enough for their convenience. The surveys were self-reported and last approximately 15 minutes per participant. An English translation of the survey is available as *Extended data* ^17^.

### Measures

 We analysed the self-reported manuscript publication as a binary outcome. Multinomial variables included gender, age, current year of career, English proficiency, courses in PubMed, courses in Scopus, courses in Scielo and provider of pirated academic accounts. Binary variables included university, affiliation to a Scientific Medical Student Society, studied previous career, courses in scientific data bases, courses in scientific writing, courses in scientific browsing, courses in Zotero, use of Sci-hub, use of pirated academic accounts. All of these variables were assessed with a self-administered survey.

### Data analysis

We evaluated the association between self-reported manuscript publication and its covariates using chi-squared tests for categorical variables and Mann-Whitney U-test for numerical variables. Poisson family regressions were performed using a log link function and mixed effects multilevel models. We estimated nested models following a manual forward selection method to identify covariates associated with self-reported manuscript publication until reaching a parsimonious multivariable model. These covariates were selected using likelihood ratio tests. Crude and adjusted prevalence ratios (PR) were estimated with 95% confidence intervals (CI 95%). All hypotheses were contrasted using 5% significance. The analysis was performed using Stata 15.1, and the analysis code is openly available on GitHub and Zenodo ^18^.

### Ethical considerations

This study was classified as minimal risk for participants by the San Bartolome Hospital’s Institutional Review Board (CIE15325-15) and issued its approval. Trained interviewers verbally consent participants, provided them an anonymized self-administered survey. Also, each survey was assigned a numeric ID to protect the privacy of the participants.

## Results

We interviewed 11,587 medical students, of whom the mean age was 21±2.9 years and 53% were female. In total, 22.2% (n=2,575) of medical students were in the first year of career. There were 12.5% (n=1,449) affiliated to a Scientific Medical Student Society and 14.1% (n=1,618) reported advanced English skills. The individual-level responses are available as *Underlying data* ^19^.

A total of 65.1% (n=3,989) had attended a scientific writing course, and 7.9% (n=893) published at least one scientific article during his medical training. There were 22.6% (n=2,514) with a pirated academic account, and from a total of 6,632 medical students, 19.2% (n=1,273) used Sci-Hub at a certain point of their careers ( Table 1).

**Table 1.** Characteristics of medical students from 40 schools of medicine in Latin America.

Characteristics	N=11,587	n	%
Gender	11,587		
Male		5,363	46.3
Female		6,224	53.7
Age (years) ^*^		21±2.86	
University	11,587		
National		6,119	52.8
Private		5,468	47.2
Current year of career	11,586		
1 ^st^		2,575	22.2
2 ^nd^		2,486	21.5
3 ^rd^		2,053	17.7
4 ^th^		1,969	17.0
5 ^th^		1,585	13.7
6 ^th^		918	7.9
Affiliated to a Scientific Medical Student Society	11,587		
No		10,138	87.5
Yes		1,449	12.5
English proficiency	11,499		
Elementary		2,028	17.6
Basic		4,666	40.6
Intermediate		3,187	27.7
Advanced		1,618	14.1
Studied previous career	11,574		
No		10,689	92.4
Yes		885	7.7
Courses in scientific databases	11,448		
No		5,300	46.3
Yes		6,148	53.7
Courses in PubMed	11,297		
Do not use the database		4,529	40.1
No		3,686	32.6
Yes		3,082	27.3
Courses in Scopus	11,139		
Do not use the database		9,334	83.8
No		896	8.0
Yes		909	8.2
Courses in Scielo	11,200		
Do not use the database		4,918	43.9
No		4,165	37.2
Yes		2,117	18.9
Courses in scientific writing	11,417		
No		7,428	65.1
Yes		3,989	34.9
Courses in scientific searches	11,458		
No		4,564	39.8
Yes		6,894	60.2
Courses in Zotero	11,408		
No		9,485	83.1
Yes		1,923	16.9
Use of Sci-Hub	6,632		
No		5,359	80.8
Yes		1,273	19.2
Pirated academic accounts	11,136		
No		8,622	77.4
Yes		2,514	22.6
Provider of pirated academic accounts	11,063		
Student		1,751	15.8
Professor		1,817	16.4
Both		14	16.4
Do not answer		7,481	67.6
Scientific publication	11,316		
No		10,423	92.1
Yes		893	7.9

^*^ Mean ± standard deviation.

There were differences on the prevalence of scientific publications among first-year and last-year medical students (13% vs 4.3%, respectively), being affiliated to a Scientific Medical Student Society (12.43% vs 7.24%), the advanced and elementary proficiency of English (11.2% vs 6.4%), took a scientific writing course (14.6% vs 4.3%), use Sci-Hub (19.3% vs 4.7%) and having pirated academic accounts (15.3% vs 5.5%) ( Table 2).

**Table 2.** Characteristics of medical students among scientific publication from 40 schools of medicine in Latin America.

Characteristics	Scientific publication				*P* value
	No		Yes		
	n	%	n	%	
Gender					0.529
Male	4,823	91.9	423	8.1	
Female	5,600	92.3	470	7.7	
Age (years) *	0.99				<0.001
University					<0.001
National	5,389	90.2	589	9.9	
Private	5,034	94.3	304	5.7	
Current year of career					<0.001
1 ^st^	2,417	95.7	108	4.3	
2 ^nd^	2,258	92.9	172	7.1	
3 ^rd^	1,855	92.3	155	7.7	
4 ^th^	1,763	90.9	177	9.1	
5 ^th^	1,382	89.1	169	10.9	
6 ^th^	748	87.0	112	13.0	
Affiliated to a Scientific Medical Student Society					<0.001
No	9,176	92.8	716	7.24	
Yes	1,247	87.6	177	12.43	
English proficiency					<0.001
Elementary	1,869	93.6	127	6.4	
Basic	4,240	93.1	315	6.9	
Intermediate	2,869	91.4	271	8.6	
Advanced	89	88.8	175	11.2	
Studied previous career					<0.001
No	9,684	92.7	765	7.3	
Yes	729	85.2	127	14.8	
Courses in scientific databases					<0.001
No	5,024	96.3	195	3.7	
Yes	5,356	88.5	697	11.5	
Courses in PubMed					<0.001
Do not use the database	4,220	94.2	259	5.8	
No	3,283	91.2	317	8.8	
Yes	2,720	89.8	309	10.2	
Courses in Scopus					<0.001
Do not use the database	8,531	92.7	673	7.3	
No	795	89.6	92	10.4	
Yes	798	88.4	105	11.6	
Courses in Scielo					<0.001
Do not use the database	4,624	95.3	226	4.66	
No	3,634	88.5	472	11.5	
Yes	1,905	91.5	178	8.55	
Courses in scientific writing					<0.001
No	6,993	95.7	317	4.34	
Yes	3,366	85.4	574	14.57	
Courses in scientific browsing					<0.001
No	4,298	95.4	206	4.57	
Yes	6,091	89.9	684	10.1	
Courses in Zotero					<0.001
No	8,774	93.9	570	6.1	
Yes	1,579	83.2	320	16.85	
Use of Sci-Hub					<0.001
No	5,026	95.3	246	4.67	
Yes	1,016	80.7	243	19.3	
Pirated academic accounts					<0.001
No	8,055	94.5	468	5.49	
Yes	2,102	84.7	380	15.31	
Provider of pirated academic accounts					<0.001
Student	1,486	86.1	242	3.28	
Professor	1,453	80.3	239	13.86	
Both	12	85.7	357	19.72	
Do not answer	7,140	96.7	2	14.29	

* Simple logistic regression. Beta and p-value.

The nested models progressively selected the following covariates: courses in scientific writing, pirated academic accounts, universities, courses in Zotero, courses in scientific databases, year of study, previous career, English proficiency, and affiliation to a scientific medical student society. The prevalence of having a scientific publication were 36% (PRp=1.36, 95%CI=1.16–1.59, p<0.001) higher if a medical student was affiliated to a Scientific Medical Student Society, 51% (PRp=1.51, 95%CI=1.21–1.87, p<0.001) higher among the medical students with advanced English proficiency, 85% (PRp=1.85, 95%CI=1.59–2.15, p<0.001) higher in medical students who took a scientific writing course, 81% (PRp=1.81, 95%CI=1.50–2.20, p<0.001) higher in medical students who used Sci-Hub, and 108% (PRp=2.08, 95%CI=1.83–2.36, p<0.001) higher among medical students who have a pirated academic account ( Table 3). The information about medical schools in Latin America is available as *Extended data* ^20^.

**Table 3.** Associated factors with scientific publication among medical students from 40 schools of Medicine in Latin America and the Caribbean.

Parameters	Scientific publication															Models**
	Simple regression (1)					Multiple regression parsimonious model (2)					Adjusted parsimonious model (2) *					
	PRc	95% CI			*P* value	PRp	95% CI			*P* value	PRp	95% CI			*P* value	
Gender																1
Male	Ref.										Ref.					
Female	0.96	0.85	-	1.09	0.529						0.99	0.87	-	1.12	0.865	
Age (years) *											1.02	0.99	-	1.045	0.305	2
University																
National	1.73	1.51	-	1.98	<0.001	1.82	1.58	-	2.09	<0.001						
Private	Ref.					Ref.										
Current year of career																
1 ^st^	Ref.					Ref.										
2 ^nd^	1.65	1.31	-	2.09	<0.001	1.27	1.01	-	1.60	0.043						
3 ^rd^	1.80	1.42	-	2.29	<0.001	1.39	1.09	-	1.76	0.007						
4 ^th^	2.13	1.69	-	2.69	<0.001	1.29	1.02	-	1.64	0.036						
5 ^th^	2.55	2.02	-	3.22	<0.001	1.64	1.30	-	2.07	<0.001						
6 ^th^	3.04	2.36	-	3.92	<0.001	1.79	1.38	-	2.33	<0.001						
Affiliated to a Scientific Medical Student Society																
No	Ref.					Ref.										
Yes	1.72	1.47	-	2.00	<0.001	1.36	1.16	-	1.59	<0.001						
English proficiency																
Elementary	Ref.					Ref.										
Basic	1.09	0.89	-	1.33	0.412	1.11	0.91	-	1.35	0.302						
Intermediate	1.36	1.11	-	1.66	0.003	1.22	0.99	-	1.49	0.057						
Advanced	1.76	1.42	-	2.19	<0.001	1.51	1.21	-	1.87	<0.001						
Studied previous career																
No	Ref.					Ref.										
Yes	2.03	1.70	-	2.41	<0.001	1.68	1.41	-	2.00	<0.001						
Courses in scientific databases																
No	Ref.					Ref.										
Yes	2.21	1.90	-	2.57	<0.001	1.58	1.33	-	1.88	<0.001						
Courses in PubMed																3
Do not use the database	Ref.											Ref.				
No	1.52	1.30	-	1.78	<0.001						0.96	0.81	-	1.12	0.582	
Yes	1.76	1.51	-	2.07	<0.001						0.86	0.72	-	1.01	0.069	
Courses in Scopus																4
Do not use the database	Ref.										Ref.					
No	1.42	1.15	-	1.74	0.001						1.02	0.82	-	1.27	0.848	
Yes	1.59	1.31	-	1.93	<0.001						0.92	0.76	-	1.13	0.427	
Courses in Scielo																5
Do not use the database	Ref.										Ref.					
No	2.47	2.12	-	2.87	<0.001						1.47	1.24	-	1.74	<0.001	
Yes	1.83	1.52	-	2.22	<0.001						0.95	0.77	-	1.18	0.644	
Courses in scientific writing																
No	Ref.					Ref.										
Yes	3.36	2.95	-	3.83	<0.001	1.85	1.59	-	2.15	<0.001						
Courses in scientific searches																
No	Ref.															
Yes	3.08	2.64	-	3.60	<0.001											
Courses in Zotero																
No	Ref.					Ref.										
Yes	2.76	2.43	-	3.14	<0.001	1.66	1.45	-	1.90	<0.001						
Use of Sci-Hub																6
No	Ref.										Ref.					
Yes	4.14	3.50	-	4.88	<0.001					1.81	1.50	-	2.20	<0.001		
Pirated academic accounts																
No	Ref.					Ref.										
Yes	2.79	2.45	-	3.17	<0.001	2.08	1.825	-	2.36	<0.001						
Provider of pirated academic accounts																7
Student	4.23	3.56	-	5.01	<0.001						3.86	3.05	-	4.90	<0.001	
Professor	6.02	5.15	-	7.02	<0.001					4.56	3.85	-	5.39	<0.001		
Both	4.36	1.20	-	15.82	0.025						3.83	0.93	-	15.69	0.062	
Do not answer	Ref.										Ref.					

(1) Poisson´s regression model with robust variance. (2) Poisson´s regression model with robust variance and multilevel analysis. * Multiple regression parsimonious model was indepently adjusted by each variable below Abbreviations: PRc, Crude prevalence ratio; PRp, Parsimonious model's prevalence ratio; PRa, adjusted parsimonious' prevalence ratio.

## Discussion

### Pirated academic accounts and use of Sci-Hub

 The use of Sci-Hub was reported by 19.2% (n= 1273) of the students surveyed, of whom 19.3% (n=243) published a manuscript during their medical training. The awareness and use of Sci-Hub may be due to the high need to access top-level scientific evidence behind a paywall. However, medical students have reported difficulties to access Sci-Hub because it is considered an illegal service in many regions, meaning the web domain is often blocked ^21– 24^.

 The use of Sci-Hub was associated with higher prevalence of scientific publications among medical students (PR: 1.81; CI95%: 1.50-2.20). Students feel the great need to obtain access to payed articles, leading them to seek free access throughout Sci-Hub ^23, 25^. However, even those students who do not face a paywall, found using Sci-Hub reduced the time and increased simplicity of browsing ^26^. In addition, many researchers and students identify Sci-Hub as a faster option that is not limited to their institution's catalogue ^24^. This is likely homogeneous between high- and low-income countries worldwide ^27^. More than 56,000 article downloads via Sci-Hub came from different east coast cities of the United States, especially from cities where large universities have subscriptions to different publishers ^26^.

 The use of pirated academic accounts was associated with higher prevalence of scientific publications (PR: 2.08; CI95%: 1.83–2.36). The institutional licenses let access to journals, books, or specialized databases such as Scopus or Web of Science. These paid services are funded by government institutions in low- or middle-income countries (LMIC); however, these are not widely distributed or have not been implemented in LMIC ^28^. Alternatives such as HINARI allow access to paid articles in LMIC, and is available to the academic and research community only from certified institutions who achieved certain milestones defined by local science systems ^29^. All this complex context leads users to exchange, loan or acquire access accounts or proxy links to journal catalogs of institutions by non-legal terms ^27^.

### Courses in scientific writing

 A total of 34.9% of students who had produced a scientific publication attended a course in scientific writing skills. Attending a scientific writing skills course increased the prevalence of scientific publications by 85% (PRp=1.85, CI95%=1.59–2.15, p<0.001). This is likely because of the great need of medical students to improve their skills to effectively communicate scientific findings, make a relevant academic reflection, and enhance the chances of acceptance into a scientific journal ^30^. New medical students in research training are eager to be trained in scientific writing skills and seek an experienced mentor to train them ^31^. In addition, medical students actively seek for courses of scientific writing and communication, for instance, the Brazilian DivulgaMicro initiative was a course funded by the Fundação de Amparo a Pesquisa do Estado de São Paulo (FAPESP, by its acronym in Portuguese) to train early career researchers to translate complex scientific messages to understandable pieces of information to community members ^32^. At 30 days after launch, the website registered 1,026 users from different regions worldwide, including Latin American, United Kingdom, Pakistan, Germany and Canada. This was one of the most visited free and open scientific communication workshops, which trained over 600 novel medical student researchers ^32^.

### English proficiency

An advanced English proficiency was reported in 14.1% of the students, of whom 11.2% published a scientific manuscript during medical training. In addition, the prevalence of scientific publications increased by 51% among students with advanced English proficiency (PRp=1.51, 95%CI=1.21–1.87, p<0.001). Students are encouraged to understand a scientific evidence written in English ^33^. The TOEFL score is correlated with publishing in a medical journal (correlation coefficient: 0.63) ^34^. Scientific journals preferably accept articles from English natives versus non-English natives (acceptance rate 7% vs 3.6%, respectively) ^35^. Likewise, Americans are 49% more likely to reach an article peer-review or acceptance in an American journal comparing to non-English natives ^35^. Medical students from second to sixth year who attended an English scientific writing skills training reported 53% of them perceived that they were not proficient enough at English to publish a manuscript in English-language journals ^36^.

The association between scientific publications and advanced English proficiency could be likely because of students’ desire to pursue an academic training abroad offered by institutions requiring academic excellence and a great potential. In 2016, the Peruvian Program of Educational Grants and Credits (PRONABEC, by its acronym in Spanish) jointly funded the Fulbright, FONDECYT, and Chevening scholarships in Peru which benefited 14, 6, and 15 Peruvian graduate applicants, respectively ^37^. In this way, the scholars could be trained by outstanding foreign universities producing a generation of researchers with masters and doctoral degrees who upon returning to their home countries seek to improve the science and technology system ^38– 40^. During 2004–2012, the Fogarty International Clinical Research Fellows Program funded promising initiatives of highly competitive English-dominant students from LMIC whose scientific discoveries can address long-term global health needs ^41, 42^. This approach has become Fogarty's hallmark: bringing great science to solve local problem of global outreach and building local research capacities ^42^. During 2014–2015, Fogarty has contributed substantially to the training of more than 6,100 global health leaders, 140 of whom have earned doctorates in epidemiology and 96 in public health ^43^.

The Fogarty International Center builds a bridge between the US National Institutes of Health and the global health research community; 85–90% of trained fellows return to LMICs and obtain research positions into academia, government agencies, and institutes ^42^. However, young Latin American scholars and postdoctoral researchers trained abroad find it difficult because of an unfavourable science system ^29^. For instance, the investment of the Peruvian administration to progress in science and research is still insufficient, it is only 0.12% of the gross domestic product compared to 0.36% in Chile, 1.3% in Brazil and 2.8% in the United States ^44, 45^.

### Affiliated to a scientific medical student society

 Our findings showed that being affiliated to a medical student scientific society increased the prevalence of scientific publication in 36% (PRp:1.36, CI95%=1.16-1.59, p<0.01). Student scientific societies, such as the Peruvian Medical Student Scientific Society (SOCIMEP, by its acronym in Spanish) tries to fill the gaps of research training and provide students the mentors, courses, and scientific opportunities to pursue a research career ^9, 46^. With over 30 years of operations with local-level scientific societies across Peru, SOCIMEP promotes research events at a regional, national, and local level multidisciplinary university research and service camps (CUMIS, by its acronym in Spanish), the annual scientific conferences, and foundation courses in epidemiology, research design, and biostatistics ^47^. The outreach the SOCIMEP achieved overall was reflected in the 242 published articles by scientific societies, of which 11% (n=67) were published in Q1 journals, under the mentorship of highly experienced national researchers ^48^.

### Limitations

We must understand our findings have limitations, described in the following statements. First, several parameters of the questionnaire were self-reported which may cause an undifferentiated classification of the outcome, and may increase the residual confusion of confounding parameters, indicating potential information bias. However, we tried to control this situation by motivating the students to answer the questionnaire truthfully and not to rush their answers; in this sense, our outcome is consistent with reality. Second, all 40 medical schools were affiliated with FELSOCEM, indicating potential selection bias, so our findings are useful for these schools and similar studies should be extended to understand local and regional scientific realities from different countries.

## Conclusion

 Factors associated with producing a publication among medical students during their medical training in Latin America include being affiliated to a local Scientific Society of Medical Students, having an advanced English proficiency, attended a course of scientific writing skills, the use of Sci-Hub, and having pirated accounts. The promotion of science among medical students in Latin America is a multi-sphere issue which needs to be addressed as part of multilevel strategies coming from high government authorities, to empower universities and build-up a committed science system in each nation.

## Data availability

### Underlying data

Figshare: Scientific article. https://doi.org/10.6084/m9.figshare.13061699.v2 ^19^.

This project contains the underlying data in DTA and CSV formats.

### Extended data

Figshare: Technological and educational factors associated with the use of information sources in medical students from Latin America. https://doi.org/10.6084/m9.figshare.13070603.v1 ^17^.

This project contains an English-language copy of the questionnaire used for data collection.

Figshare: Latin American medical students surveyed in 2016 - Supplementary materials from a cross-sectional study of 40 medical schools surveyed in Latin America. https://doi.org/10.6084/m9.figshare.13070693.v1 ^20^.

This project contains a list of the medical schools surveyed for this study.

Analysis code used in this study is available at: https://github.com/culquichicon/Scientific_writing.

Archived code at time of publication: https://doi.org/10.5281/zenodo.3730359 ^18^.

Analysis code license: GNU General Public License v3.0.

Unless otherwise indicated, data are available under the terms of the Creative Commons Attribution 4.0 International license (CC-BY 4.0).
