# Factors associated with producing a scientific publication during medical training: evidence from a cross-sectional study of 40 medical schools in Latin America

**DOI:** 10.12688/f1000research.26596.2

**Published:** 2022-05-23

**Authors:** Mario J. Valladares-Garrido, Christian R. Mejia, Annel B. Rojas-Alvarado, Mary M. Araujo-Chumacero, Jhacksson S. Córdova-Agurto, Jessica Fiestas, Feeder J. Rojas-Vilar, Carlos Culquichicón

**Affiliations:** 1 Vicerrectorado de Investigación, Universidad Norbert Wiener, Lima, Peru; 2 Instituto de Evaluación de Tecnologías en Salud e Investigación-IETSI, EsSalud, Lima, Peru; 3 Universidad Continental, Lima, Peru; 4 School of Medicine, Universidad Privada Antenor Orrego, Piura, Peru; 5 CI- Emerge, Center of Emerging Diseases and Climate Change, Universidad Nacional de Piura, Piura, Peru; 6 Scientific Society of Medical Students, Universidad Privada Antenor Orrego, Piura, Peru; 7 School of Health Sciences, Universidad Nacional de Piura, Piura, Peru; 8 School of Medicine, Universidad Cesar Vallejo, Piura, Peru; 9 Facultad de Medicina, Universidad Ricardo Palma, Lima, Peru; 10 Universidad Privada Norbert Wiener, Centro de Investigación Epidemiológica en Salud Global, Lima, Peru

**Keywords:** Medical Education, Undergraduate, Scientific Societies, Latin America, Medical Students

## Abstract

**Background: **Scientific publication during medical training is key to promoting enduring cutting-edge knowledge. The promotion of science among medical students in Latin America is a multisectoral issue that is hampered by the lack of governmental knowledge to invest in national research, as well as by the lack of support from local universities. This study aims to determine the factors associated with the production of a scientific publication during medical training among Latin American medical students of local scientific societies.

**Methods: **This is a secondary data analysis of a cross-sectional study conducted in 2016 that assessed the use of information and communication technologies (ICTs) among medical students from 40 local scientific societies of medical students affiliated with FELSOCEM. Teams from each local scientific society surveyed self-reported scientific publications and explored their association with socioeconomic, academic, and research training conditions. We applied nested models to identify the covariates associated with self-reported scientific publication, obtaining a parsimonious mixed-effects multilevel model grouped by medical scientific society.

**Results: **Of 11,587 participants, the prevalence of scientific publications increased in 36% among medical students affiliated to a Scientific Society of Medical Students [parsimonious prevalence ratio (pPR)=1.36, 95%CI=1.16–1.59], 51% among medical students with advanced English proficiency [pPR=1.51, 95%CI=1.21 – 1.87], 85% among medical students who attended a scientific writing skills course [pPR=1.85, 95%CI=1.59–2.15], 81% among medical students who use Sci-Hub [pPR=1.81, 95%CI=1.50–2.20], and 108% among medical students who have access to a pirated academic account [pPR=2.08, 95%CI=1.83–2.36].

**Conclusions: **Producing a scientific publication among medical students is associated with being affiliated to a scientific society of medical students, English proficiency, training in scientific writing, use of Sci-Hub, and pirated academic accounts. The results will help clinical educators and medical programs improve resources for training students in high-quality research

## Introduction

Producing a scientific publication during medical training is key to promoting continuing medical education and encouraging trainees to create cutting-edge knowledge. In doing so, students will develop research and critical thinking skills and will carry out evidence-based practice and patient-centered care with an enduring vision for pursuing a scientific career ^1– 3^. Latin American universities are progressively recognizing the critical importance of fostering science at the beginning of the bachelor's degree, and are implementing research-oriented courses such as research design methods, biostatistics, epidemiology, and a research-focused thesis ^4^. However, there are still gaps in Latin America compared to university research systems in developed countries in terms of number of publications, quality of published articles, dissemination of studies, and funding opportunities ^5^. Studies in Colombia and Brazil show that medical students consider scientific research as an important aspect of their training and that the low scientific output is influenced by the lack of inspiring and committed mentors as role models for the beginning of the scientific career ^6, 7^. Between 1997 and 2010, there was an 8.4% increase in student participation in manuscripts published in journals indexed in Scielo-Peru, of which 42% reported being affiliated with a medical student scientific society ^4, 8^.

In Peru, the progress of undergraduate medical research has been strongly promoted by the Peruvian Scientific Society of Medical Students (SOCIMEP, by its acronym in Spanish), an organization that has been improving the research training of medical students for 27 years ^9^. SOCIMEP is organized in scientific and academic committees and is made up of 38 local scientific societies in all Peruvian medical schools. This society is recognized for the organization of international, national, and local scientific conferences ^9^. SOCIMEP also encourages the active participation of societies and integrates them into a nationwide research network, and provides connections to experienced research mentors. Being affiliated to a local scientific society affiliated to SOCIMEP is associated with a higher scientific production (PR: 2.41; 95% CI: 1.55-3.74) ^10^. However, only 10% of the projects carried out in local scientific societies are published in indexed journals due to poor methods applied in the studies, lack of knowledge of the editorial process, few local mentors, and lack of financial support from public agencies and institutions ^11^. Funding opportunities for medical students are scarce in local medical schools in Peru and in much of Latin America. Overall government investment is disproportionately granted and often contradictory to local public health needs, detracting from the importance of well-implemented laboratories and full-time research-focused faculties ^12^. In Peru, less than 30% of universities have funding programs for students to conduct thesis research, or awards for student research programs ^13^.

The promotion of science among medical students in Latin America is a multi-sectoral issue that is hampered by governments' lack of knowledge about investing in well-structured national research and innovation systems, as well as by the lack of support from local universities, their lack of investment in research facilities, and the lack of mentors with international research experience ^3^. Improvement of the scientific system in Latin America might be valuable for other regions of the world, by promoting high-quality research at the undergraduate-level, an integrative ecosystem of research and education would be consolidated for to better medical practice, and training of health professionals in similar settings across the globe. In this scenario, our study aims to determine the factors associated with scientific publication during medical training, in order to identify the needs of local Latin American scientific societies for the implementation of continuing education programs in research. Our hypothesis is that there are factors in medical training associated with the production of a scientific publication during undergraduate training.

## Methods

### Study design

This is a secondary data analysis of a cross-sectional study initially conducted in 2016 to assess the use of information and communication technologies (ICTs) in medical students across Latin America ^14, 15^. This study evaluated 40 medical student scientific societies in Latin America. The self-report of having a scientific publication was evaluated. In addition, the following variables were used to explore factors associated with scientific publication: gender, age, university, current year of study, medical student scientific society membership, English proficiency, previous career studied, courses in scientific databases including PubMed, Scopus and Scielo, courses in scientific writing skills, courses in scientific navigation, courses in Zotero, use of Sci-Hub, and access to and provision of pirated academic accounts.

### Population and sampling

The primary study surveyed 11,587 students from 40 medical schools, including two from Ecuador, two from Panama, four from Paraguay, three from Bolivia, 18 from Peru, two from Mexico, two from Venezuela, one from Honduras, three from Colombia, one from Chile and two from Argentina. Medical students enrolled in the 2016-I term were included in this study and those doing their internship were excluded.

We performed stratified sampling using the academic year in medical school as the stratum. The estimated sample size for each research center was 289 medical students, to which 10% was added to allow for dropouts. Thus, we set out to survey 318 medical students at each university. We considered a sample size calculation with 80% power and 5% significance for an infinite sample size. The use of these parameters is a convention to determine a conservative sample size that can detect a minimal difference for the outcome. As for the selection of the participants, the interview team went into the course with the highest credit in each academic year and chose the students who were seated in an odd place per row. In three universities, the sample size was not large enough to reach the minimum required, so we surveyed to all students.

### Operational procedures

In 2015, the ICTs project was awarded an amount of money for publication in the multicenter project competition of the 30th International Congress of the Latin American Federation of Scientific Societies of Medical Students (FELSOCEM). This award allowed the authors to contact the researchers of the FELSOCEM international collaboration network for the development of the study. We were able to register teams from 40 out of 69 Scientific Societies of Medical Students (SOCEM) throughout Latin America. Each scientific society had at least one team with three medical students who received training on scientific integrity ^16^, standardized methods for survey participants, data entry procedures and quality control of the datasets.

In each medical school, a designated team of interviewers surveyed at the beginning or end of lectures, prioritizing that students had enough time for their comfort. The questionnaire was given to each selected student after explaining the objective of the study and the duration of the survey (approximately 15 minutes). The survey was self-reported, that is, the participants provided the answers themselves. An English translation of the survey is available as *Extended data* ^17^.

### Measures

Self-reporting of manuscript publication was analyzed as a binary outcome. Multinomial variables included gender, age, current year of degree, English proficiency, courses in PubMed, courses in Scopus, courses in Scielo, and provision of pirated scholarly accounts. Binary variables included university, affiliation with a medical student scientific society, previously studied career, scientific database courses, scientific writing courses, scientific navigation courses, Zotero courses, Sci-hub usage, and pirated academic account usage. All these variables were self-reported.

Sci-hub usage is defined as the use of the web service to read and download restricted scientific articles that are typically paid or subscription-linked at academic institutions. Use of pirated academic accounts is the use of any account provided by a teacher, student or other person that helps the student find and download articles from academic institutions that subscribe to scientific journals or databases.

### Data analysis

The association between self-reporting of manuscript publication and its covariates was assessed using chi-square tests for categorical variables and the Mann-Whitney U test for numerical variables. Poisson family regressions were performed using a log link function and mixed effects multilevel models. Nested models were estimated following a forward manual selection method using likelihood ratio tests. Covariates with significant p-values (p < 0.05) were included in the further nested model until statistical significance was not reached. This method was used to obtain a parsimonious multivariate model, which retains the least amount of covariates to explain the variance of the outcome. Crude and adjusted prevalence ratios (PR) were estimated with 95% confidence intervals (95%CI). All hypotheses were tested with a significance of 5%. The analysis was performed using Stata 15.1. The code is openly available on GitHub and Zenodo ^18^.

### Ethical considerations

This study was classified as minimal risk for participants by the Institutional Review Board of San Bartolome's Hospital (CIE15325-15), and issued its approval. Trained interviewers obtained verbal consent from participants and provided them with an anonymous self-administered survey. Each survey was assigned a numerical ID to protect the privacy of the participants.

## Results

A total of 11,587 medical students completed the survey. The mean age was 21±2.9 years, 53% were female, 12.5% (n=1,449) were affiliated with a medical student scientific society, and 14.1% (n=1,618) reported advanced English language skills. The individual-level responses are available as *Underlying data* ^19^.

Scientific writing courses were attended by 65.1% (n=3,989) of the students, and 7.9% (n=893) had published at least one scientific article during their medical training. Out of 6,632 students, 19.2% (n=1,273) used Sci-Hub at some point in their career ( Table 1).

**Table 1.** Characteristics of medical students from 40 schools of medicine in Latin America.

Characteristics	N=11,587	n	%
Gender	11,587		
Male		5,363	46.3
Female		6,224	53.7
Age (years) ^*^		21±2.86	
University	11,587		
National		6,119	52.8
Private		5,468	47.2
Current year of career	11,586		
1 ^st^		2,575	22.2
2 ^nd^		2,486	21.5
3 ^rd^		2,053	17.7
4 ^th^		1,969	17.0
5 ^th^		1,585	13.7
6 ^th^		918	7.9
Affiliated to a Scientific Medical Student Society	11,587		
No		10,138	87.5
Yes		1,449	12.5
English proficiency	11,499		
Elementary		2,028	17.6
Basic		4,666	40.6
Intermediate		3,187	27.7
Advanced		1,618	14.1
Studied previous career	11,574		
No		10,689	92.4
Yes		885	7.7
Courses in scientific databases	11,448		
No		5,300	46.3
Yes		6,148	53.7
Courses in PubMed	11,297		
Do not use the database		4,529	40.1
No		3,686	32.6
Yes		3,082	27.3
Courses in Scopus	11,139		
Do not use the database		9,334	83.8
No		896	8.0
Yes		909	8.2
Courses in Scielo	11,200		
Do not use the database		4,918	43.9
No		4,165	37.2
Yes		2,117	18.9
Courses in scientific writing	11,417		
No		7,428	65.1
Yes		3,989	34.9
Courses in scientific searches	11,458		
No		4,564	39.8
Yes		6,894	60.2
Courses in Zotero	11,408		
No		9,485	83.1
Yes		1,923	16.9
Use of Sci-Hub	6,632		
No		5,359	80.8
Yes		1,273	19.2
Pirated academic accounts	11,136		
No		8,622	77.4
Yes		2,514	22.6
Provider of pirated academic accounts	11,063		
Student		1,751	15.8
Professor		1,817	16.4
Both		14	16.4
Do not answer		7,481	67.6
Scientific publication	11,316		
No		10,423	92.1
Yes		893	7.9

^*^ Mean ± standard deviation.

There were differences in the prevalence of scientific publications among first- and final-year medical students (4.3% first year vs. 13% final year), membership in a medical student scientific society (12.43% yes vs. 7, 24% no), advanced and elementary English proficiency (11.2% advanced vs. 6.4% elementary), completion of a scientific writing course (14.6% yes vs. 4.3% no), use of Sci-Hub (19.3% yes vs. 4.7% no) and possession of pirated academic accounts (15.3% yes vs. 5.5% no) ( Table 2).

**Table 2.** Characteristics of medical students among scientific publication from 40 schools of medicine in Latin America.

Characteristics	Scientific publication				*P* value
	No		Yes		
	n	%	n	%	
Gender					0.529
Male	4,823	91.9	423	8.1	
Female	5,600	92.3	470	7.7	
Age (years) *	0.99				<0.001
University					<0.001
National	5,389	90.2	589	9.9	
Private	5,034	94.3	304	5.7	
Current year of career					<0.001
1 ^st^	2,417	95.7	108	4.3	
2 ^nd^	2,258	92.9	172	7.1	
3 ^rd^	1,855	92.3	155	7.7	
4 ^th^	1,763	90.9	177	9.1	
5 ^th^	1,382	89.1	169	10.9	
6 ^th^	748	87.0	112	13.0	
Affiliated to a Scientific Medical Student Society					<0.001
No	9,176	92.8	716	7.24	
Yes	1,247	87.6	177	12.43	
English proficiency					<0.001
Elementary	1,869	93.6	127	6.4	
Basic	4,240	93.1	315	6.9	
Intermediate	2,869	91.4	271	8.6	
Advanced	89	88.8	175	11.2	
Studied previous career					<0.001
No	9,684	92.7	765	7.3	
Yes	729	85.2	127	14.8	
Courses in scientific databases					<0.001
No	5,024	96.3	195	3.7	
Yes	5,356	88.5	697	11.5	
Courses in PubMed					<0.001
Do not use the database	4,220	94.2	259	5.8	
No	3,283	91.2	317	8.8	
Yes	2,720	89.8	309	10.2	
Courses in Scopus					<0.001
Do not use the database	8,531	92.7	673	7.3	
No	795	89.6	92	10.4	
Yes	798	88.4	105	11.6	
Courses in Scielo					<0.001
Do not use the database	4,624	95.3	226	4.66	
No	3,634	88.5	472	11.5	
Yes	1,905	91.5	178	8.55	
Courses in scientific writing					<0.001
No	6,993	95.7	317	4.34	
Yes	3,366	85.4	574	14.57	
Courses in scientific browsing					<0.001
No	4,298	95.4	206	4.57	
Yes	6,091	89.9	684	10.1	
Courses in Zotero					<0.001
No	8,774	93.9	570	6.1	
Yes	1,579	83.2	320	16.85	
Use of Sci-Hub					<0.001
No	5,026	95.3	246	4.67	
Yes	1,016	80.7	243	19.3	
Pirated academic accounts					<0.001
No	8,055	94.5	468	5.49	
Yes	2,102	84.7	380	15.31	
Provider of pirated academic accounts					<0.001
Student	1,486	86.1	242	3.28	
Professor	1,453	80.3	239	13.86	
Both	12	85.7	357	19.72	
Do not answer	7,140	96.7	2	14.29	

* Simple logistic regression. Beta and p-value.

The nested models progressively selected the following covariates: scientific writing courses, pirated academic accounts, universities, Zotero courses, scientific database courses, year of study, previous degree, English proficiency, and medical student scientific society membership. The prevalence of having a scientific publication was 85% (pPR=1.85, 95%CI=1.59–2.15, p<0.001) higher in students who took a scientific writing course, 81% (pPR=1.81, 95%CI=1.50–2.20, p<0.001) higher for students who used Sci-Hub, and 108% (pPR=2.08, 95%CI=1.83–2.36, p<0.001) higher among students who had a pirated academic account ( Table 3). Information about medical schools in Latin America is available as *Extended data* ^20^.

**Table 3.** Associated factors with scientific publication among medical students from 40 schools of Medicine in Latin America and the Caribbean.

Parameters	Scientific publication															Models**
	Simple regression (1)					Multiple regression parsimonious model (2)					Adjusted parsimonious model (2) *					
	PRc	95% CI			*P* value	PRp	95% CI			*P* value	PRp	95% CI			*P* value	
Gender																1
Male	Ref.										Ref.					
Female	0.96	0.85	-	1.09	0.529						0.99	0.87	-	1.12	0.865	
Age (years) *											1.02	0.99	-	1.045	0.305	2
University																
National	1.73	1.51	-	1.98	<0.001	1.82	1.58	-	2.09	<0.001						
Private	Ref.					Ref.										
Current year of career																
1 ^st^	Ref.					Ref.										
2 ^nd^	1.65	1.31	-	2.09	<0.001	1.27	1.01	-	1.60	0.043						
3 ^rd^	1.80	1.42	-	2.29	<0.001	1.39	1.09	-	1.76	0.007						
4 ^th^	2.13	1.69	-	2.69	<0.001	1.29	1.02	-	1.64	0.036						
5 ^th^	2.55	2.02	-	3.22	<0.001	1.64	1.30	-	2.07	<0.001						
6 ^th^	3.04	2.36	-	3.92	<0.001	1.79	1.38	-	2.33	<0.001						
Affiliated to a Scientific Medical Student Society																
No	Ref.					Ref.										
Yes	1.72	1.47	-	2.00	<0.001	1.36	1.16	-	1.59	<0.001						
English proficiency																
Elementary	Ref.					Ref.										
Basic	1.09	0.89	-	1.33	0.412	1.11	0.91	-	1.35	0.302						
Intermediate	1.36	1.11	-	1.66	0.003	1.22	0.99	-	1.49	0.057						
Advanced	1.76	1.42	-	2.19	<0.001	1.51	1.21	-	1.87	<0.001						
Studied previous career																
No	Ref.					Ref.										
Yes	2.03	1.70	-	2.41	<0.001	1.68	1.41	-	2.00	<0.001						
Courses in scientific databases																
No	Ref.					Ref.										
Yes	2.21	1.90	-	2.57	<0.001	1.58	1.33	-	1.88	<0.001						
Courses in PubMed																3
Do not use the database	Ref.											Ref.				
No	1.52	1.30	-	1.78	<0.001						0.96	0.81	-	1.12	0.582	
Yes	1.76	1.51	-	2.07	<0.001						0.86	0.72	-	1.01	0.069	
Courses in Scopus																4
Do not use the database	Ref.										Ref.					
No	1.42	1.15	-	1.74	0.001						1.02	0.82	-	1.27	0.848	
Yes	1.59	1.31	-	1.93	<0.001						0.92	0.76	-	1.13	0.427	
Courses in Scielo																5
Do not use the database	Ref.										Ref.					
No	2.47	2.12	-	2.87	<0.001						1.47	1.24	-	1.74	<0.001	
Yes	1.83	1.52	-	2.22	<0.001						0.95	0.77	-	1.18	0.644	
Courses in scientific writing																
No	Ref.					Ref.										
Yes	3.36	2.95	-	3.83	<0.001	1.85	1.59	-	2.15	<0.001						
Courses in scientific searches																
No	Ref.															
Yes	3.08	2.64	-	3.60	<0.001											
Courses in Zotero																
No	Ref.					Ref.										
Yes	2.76	2.43	-	3.14	<0.001	1.66	1.45	-	1.90	<0.001						
Use of Sci-Hub																6
No	Ref.										Ref.					
Yes	4.14	3.50	-	4.88	<0.001					1.81	1.50	-	2.20	<0.001		
Pirated academic accounts																
No	Ref.					Ref.										
Yes	2.79	2.45	-	3.17	<0.001	2.08	1.825	-	2.36	<0.001						
Provider of pirated academic accounts																7
Student	4.23	3.56	-	5.01	<0.001						3.86	3.05	-	4.90	<0.001	
Professor	6.02	5.15	-	7.02	<0.001					4.56	3.85	-	5.39	<0.001		
Both	4.36	1.20	-	15.82	0.025						3.83	0.93	-	15.69	0.062	
Do not answer	Ref.										Ref.					

(1) Poisson´s regression model with robust variance. (2) Poisson´s regression model with robust variance and multilevel analysis. * Multiple regression parsimonious model was indepently adjusted by each variable below Abbreviations: PRc, Crude prevalence ratio; PRp, Parsimonious model's prevalence ratio; PRa, adjusted parsimonious' prevalence ratio.

## Discussion

### Pirated academic accounts and use of Sci-Hub

Sci-Hub use was reported by 19.2% (n=1273) of the students surveyed, of whom 19.3% (n=243) published a manuscript during their medical training. Awareness and use of Sci-Hub may be due to the strong need for access to high-level scientific evidence behind a paywall. This need is often reinforced because many medical schools do not offer access to high quality scientific journals or databases. However, medical students have reported difficulties in accessing Sci-Hub because it is considered an illegal service in many regions, meaning that the web domain is often blocked ^21– 24^.

Sci-Hub use was associated with a higher prevalence of scientific publication among medical students (PR: 1.81; 95%CI: 1.50-2.20). Students feel a strong need of access to paid articles, leading them to seek free access on Sci-Hub ^23, 25^. However, even those students who do not face a paywall, found using Sci-Hub reduced the time and increased simplicity of browsing ^26^. In addition, many researchers and students identify Sci-Hub as a faster option that is not limited to their institution's catalog ^24^. This process of rapid acquisition of scientific articles offered by sci-hub is probably homogeneous among high- and low-income countries around the world ^27^. More than 56,000 article downloads through Sci-Hub come from different cities on the east coast of the United States, especially from cities where major universities subscribe to different publishers ^26^.

Use of pirated academic accounts was associated with a higher prevalence of scientific publishing (PR: 2.08; 95%CI: 1.83–2.36). Pirated accounts are an alternative to institutional licenses for obtaining access to journals, books or specialized databases such as Scopus or Web of Science. Although fee-based services are financed by governmental institutions in low- and middle-income countries (LMICs), they are not widely distributed or have not been applied in LMICs ^28^. Other alternatives, e.g., HINARI, allow access to fee-based articles in LMICs, but is available to the academic and research community only from certified institutions that have reached certain milestones defined by local science systems ^29^. This complex context leads users to exchange, lend or acquire access accounts or proxy links to institutional journal catalogs under non-legal terms ^27^.

### Courses in scientific writing

One third (34.9%) of the students who have published a scientific publication have attended a course on scientific writing. Attending a scientific writing course increased the prevalence of scientific publications by 85% (pPR=1.85, 95%CI=1.59–2.15, p<0.001). This is likely due to the great need for medical students to improve their skills to effectively communicate scientific findings, make relevant scholarly reflection and increase the chances of being accepted into a scientific journal ^30^. New medical students in research training are eager to train in scientific writing skills and are looking for an experienced mentor to train them ^31^. When research courses are not locally available in institutions, students are likely to seek training in online short-courses, for example, the Brazilian initiative DivulgaMicro was a course funded by the Fundação de Amparo a Pesquisa do Estado de São Paulo (FAPESP) to train early-career researchers to translate complex scientific messages into understandable pieces of information for members of the scientific community ^32^. Within 30 days of its launch, the website registered 1,026 users from different regions of the world, including Latin America, the United Kingdom, Pakistan, Germany and Canada. This is one of the most visited free and open science communication workshops, in which more than 600 junior medical student researchers were trained ^32^. In this context, our results may encourage medical education programmers to implement scientific writing courses as part of their curricula.

### English proficiency

An advanced level of English was achieved in 14.1% of the students, and 11.2% published a scientific manuscript during their medical training. In addition, the prevalence of scientific publications increased by 51% among students with advanced English proficiency (pPR=1.51, 95%CI=1.21–1.87, p<0.001). Students are encouraged to understand scientific evidence written in English ^33^. TOEFL score correlates with publication in a medical journal (correlation coefficient: 0.63) ^34^. Scientific journals preferentially accept articles from native English speakers over non-native English speakers (acceptance rate 7% vs 3.6%, respectively) ^35^. Also, Americans are 49% more likely to have an article reviewed or accepted in a U.S. journal compared to non-native speakers ^35^. Second- to sixth-year medical students who attended a training course on scientific writing in English reported that 53% of them perceived that they were not proficient enough in English to publish a manuscript in English-language journals ^36^. Our findings may help medical educators to train student researchers to read and write more articles in English. In addition, medical curricula could establish effective medical English courses to help improve the rate of scientific publications.

The association between scientific publications and advanced English proficiency could be due to students' desire to pursue an academic education abroad offered by institutions that demand academic excellence and high potential. In 2016, the Peruvian Program for Scholarships and Educational Credits (PRONABEC) jointly funded Fulbright, FONDECYT, and Chevening scholarships in Peru that benefited 14, 6 and 15 Peruvian graduate applicants, respectively ^37^. In this way, scholarship recipients could be trained at leading foreign universities, producing a generation of researchers with master's and doctoral degrees who, upon returning to their countries of origin, seek to improve the science and technology system ^38– 40^. During 2004-2012, the Fogarty International Clinical Research Scholars and Fellows Program funded promising initiatives by students from low- and middle-income countries with English proficiency, whose scientific discoveries may address long-term global health needs ^41, 42^. This approach has become Fogarty's hallmark: bringing great science to solve local problems with global reach and building local research capabilities ^42^. During 2014-2015, Fogarty has contributed substantially to the training of more than 6,100 global health leaders, 140 of whom have earned doctoral degrees in epidemiology and 96 in public health ^43^. The process of obtaining scholarships and future academic degrees could be improved by equalizing opportunities at the undergraduate level.

Fogarty International Center bridges U.S. National Institutes of Health with global health research community; 85-90% of trained fellows return to LMICs and obtain research positions in universities, government agencies, and institutes ^42^. However, young Latin American researchers and foreign-trained postdoctoral researchers face difficulties due to an unfavorable scientific system ^29^. For example, the Peruvian administration's investment in the advancement of science and research is still insufficient, at only 0.12% of gross domestic product compared to 0.36% in Chile, 1.3% in Brazil, and 2.8% in the United States ^44, 45^. This is a concerning situation that must be addressed at the political level to efficiently solve public health needs.

### Medical student scientific society membership

Our results showed that membership in a medical student scientific society increased the prevalence of scientific publication by 36% (pPR:1.36, 95%CI=1.16-1.59, p<0.01). Student scientific societies, such as SOCIMEP, attempt to fill the gaps in research training and provide students with the mentors, courses and scientific opportunities to pursue a research career ^9, 46^. With more than 30 years of operations with local scientific societies throughout Peru, SOCIMEP promotes regional, national and local research events (CUMIS), annual scientific congresses and foundation courses in epidemiology, research design, and biostatistics ^47^. SOCIMEP's overall reach was reflected in the 242 articles published by scientific societies, of which 11% (n=67) were published in Q1 journals, under the tutelage of highly experienced national researchers ^48^. SOCIMEP's presence in Peru demonstrates the importance of an integrated institution that could not only equalize opportunities for students, but also improve scientific production in the country. Our results suggest that this student research system could be an effective model for other similar contexts.

### Limitations

Our results have limitations that are described in the following statements. First, several questionnaire items were self-reported, which may cause outcome misclassification. This means that a participant is classified to the wrong group, e.g., a student who is proficient in English feels unskilled and their response leads them being classified as a non-proficient student. and increase the potential of information bias. However, we tried to control this situation by motivating the students to answer the questionnaire in an honest manner and not to rush them; in this sense, our result is consistent with reality. Second, all 40 medical schools were affiliated with FELSOCEM, which indicates a possible selection bias because this Latin American institution is integrated by medical schools that meet standardized parameters of undergraduate scholarly. Therefore, our results are useful for these schools but should be extended to other similar local and regional realities in different countries. Third, some other factors may be missing to better understand the medical training characteristics that may influence scientific publication. For example, the type of university (private or public), the gross national income devoted to research in each participating country, and the presence of highly qualified researchers in medical schools. However, this study provides relevant information to design new studies addressing the scientific production of medical students.

## Conclusion

Factors associated with producing a scientific publication in medical students during their medical training in Latin America are being affiliated to a scientific society of medical students, having an advanced command of English, having attended a scientific writing course, the use of Sci-Hub and the use of pirate accounts. The promotion of science among medical students in Latin America is a multisectoral issue. Its development must be addressed as part of multilevel strategies coming from the highest governmental authorities. In this way, universities would be empowered and a committed scientific system would be built in each nation.

## Data availability

### Underlying data

Figshare: Scientific article. https://doi.org/10.6084/m9.figshare.13061699.v2 ^19^.

This project contains the underlying data in DTA and CSV formats.

### Extended data

Figshare: Technological and educational factors associated with the use of information sources in medical students from Latin America. https://doi.org/10.6084/m9.figshare.13070603.v1 ^17^.

This project contains an English-language copy of the questionnaire used for data collection.

Figshare: Latin American medical students surveyed in 2016 - Supplementary materials from a cross-sectional study of 40 medical schools surveyed in Latin America. https://doi.org/10.6084/m9.figshare.13070693.v1 ^20^.

This project contains a list of the medical schools surveyed for this study.

Analysis code used in this study is available at: https://github.com/culquichicon/Scientific_writing.

Archived code at time of publication: https://doi.org/10.5281/zenodo.3730359 ^18^.

Analysis code license: GNU General Public License v3.0.

Unless otherwise indicated, data are available under the terms of the Creative Commons Attribution 4.0 International license (CC-BY 4.0).
