# Supplementary material for: Factors associated with producing a scientific publication during medical training: evidence from a cross-sectional study of 40 medical schools in Latin America
Source: F1000Res. 2022 May 23;9:1365. Originally published 2020 Nov 24. [Version 2] doi: 10.12688/f1000research.26596.2 (PMC9540009; doi:10.12688/f1000research.26596.2)
Supplement: Peer review history

# Peer review: Reviewer response for version 2

**DOI:** 10.5256/f1000research.133710.r138675

**Published:** 2022-10-06

**Authors:** Megan Anakin

**Affiliations:** 1 Otago Medical School, University of Otago, Dunedin, New Zealand

Thank you for this revised version of your article. Overall, the article has been strengthened by addressing the feedback from reviewers. Thank you for addressing all of the comments and suggestion made about the first version of your article. The revisions provide the reader with enhanced descriptions of key terminology and decisions made about the study design and procedures to enable better interpretation of the results. The revisions also provide the reader with a greater understanding of the context of the study and how the problem and findings may be relevant and applicable to their context.

Is the work clearly and accurately presented and does it cite the current literature?

Partly

If applicable, is the statistical analysis and its interpretation appropriate?

Partly

Are all the source data underlying the results available to ensure full reproducibility?

Yes

Is the study design appropriate and is the work technically sound?

Yes

Are the conclusions drawn adequately supported by the results?

Yes

Are sufficient details of methods and analysis provided to allow replication by others?

Yes

Reviewer Expertise:

My research area encompasses teaching, learning, curriculum, and faculty development in health professions education.

I confirm that I have read this submission and believe that I have an appropriate level of expertise to confirm that it is of an acceptable scientific standard.

# Peer review: Reviewer response for version 1

**DOI:** 10.5256/f1000research.29360.r128598

**Published:** 2022-04-14

**Authors:** Megan Anakin

**Affiliations:** 1 Otago Medical School, University of Otago, Dunedin, New Zealand

Review of

*Factors associated with producing a scientific publication during medical training: evidence from a cross-sectional study of 40 medical schools surveyed in Latin America*. DOI: 10.12688/f1000research.26596.1

**General comments**

 The authors provide a report that addresses the aim of a secondary data analysis to better understand the factors from medical training associated with producing a scientific publication during undergraduate study. One major recommendation is to enhance the focus of the article to emphasise the relevance, importance, and implications of study and its findings to education more generally, and to students, teachers, and medical programme curriculum and resourcing, specifically.

 Note: I appreciate that English might not be the authors' first language. I have made suggestions to improve the grammar of the manuscript. For example, in first sentence of the abstract, the verb 'promote' should be 'promoting'. I have not identified all instances of where subject-verb agreement needs checking. I recommend that a copy of the manuscript is shared with a colleague who can edit the manuscript for grammar or the authors access a professional editing service. Other grammar suggestion examples are:

- Introduction section, first paragraph, first sentence: The verb 'promote' makes more sense as 'promoting' and the verb 'endure' makes more sense as 'enduring'
- Introduction section, first paragraph, second sentence: The verb 'foster' makes more sense as 'fostering'
- Introduction section, first paragraph, third sentence: The verb 'comparing' makes more sense as 'compared'
- Introduction section, second paragraph, second sentence: Subject-verb agreement. Since the SOCIMEP is singular, then the verbs should be 'fosters' and 'provides'.

**Specific feedback with constructive comments**

**Abstract**

 The authors succinctly outline the topic, a problem that is addressed by the methods, and present the main results. Missing from the abstract is a few sentences outlining the discussion section. Please consider outlining the key discussion points related to the significance and implications of the results for clinical educators who will be reading this article.

**Introduction**

 Second paragraph, awkward word choice: I'm not certain what 'stands' means in this sentence. Please consider revising this long sentence into two shorter ones. The second sentence could begin: "SOCIMEP holds international,..."

 The introduction provides a well-argued warrant for the study by established the local need for the study. As a reader from outside Latin America, I am wondering how this situation might be similar and different to other regions. Please consider relating this problem to the locations of readers beyond Latin America.

**Methods**

 The aim is clearly stated at the beginning of this section and the methods presented are appropriate to address it.

*Population and sampling section*. Please explain the educational outcome-related evidence that was used to determine the power calculation, or if not, state the reason for why you considered a sample size calculation with an 80% of power and 5% of significance. Please describe the census-type sampling procedure or support the terminology with a reference that describes it to the reader.

*Operational procedures section*. First paragraph, first sentence: Please consider replacing XXX with number or word because it looks like information has been omitted to the reader, or please further explain this reference. Please specify what the ICT project was awarded or to whom the project was awarded. Please consider revising the second to last sentence of the second paragraph to explain how participants completed the surveys to provide responses selected or written by themselves and participants took about 15 minutes to complete it.

*Measures section*. Please clarify the term, ‘self-administered survey’ by providing more information or revising the sentence so it better matches the description of the survey in the operational procedures section.  

*Data analysis section*. The analyses performed by the authors looks appropriate, however, the explanations are dense and may not be easily understood by readers of this journal. Please revise this section so that it is suitable for clinical educators who are not necessarily biomedical scientists or statisticians. Please clarify in the text below on what statistical basis the covariates were selected so the reader does not need to make inferences about the procedures. Please explain if the forward addition assessed using a LRT and if so, explain if a chi-squared distribution or something else was used. Please also describe the criterion used to include or exclude the covariate. Otherwise, the reader has to make the assumption that this information is encompassed in the ‘all hypotheses…’ statement. Please consider stating the following information more thoroughly and with descriptions meaningful to a clinical educator reader: “We estimated nested models following a manual forward selection method to identify covariates associated with self-reported manuscript publication until reaching a parsimonious multivariable model. These covariates were selected using likelihood ratio tests. Crude and adjusted prevalence ratios (PR) were estimated with 95% confidence intervals (CI 95%). All hypotheses were contrasted using 5% significance”

**Results**

 First paragraph, first sentence: Please resolve the following contradiction: The methods state that participants completed surveys, however, the first sentence of the results states that interviews were conducted. Both cannot be true.

 Please consider revising the first, second, third, and fourth paragraphs of the results section so the information presented in them is not repeated in the tables of results as well. Instead, please draw the reader’s attention to important or relevant proportions, relationships, differences, and similarities among the results. In the third paragraph, the percentage stated for the prevalence of scientific publications among first-year and last-year medical students (13% vs 4.3%, respectively) is backwards from the Table 2. The remaining differences need to have the two categories identified. For example, the reader does not know by reading the sentence what being affiliated to a Scientific Medical Student Society (12.43% vs 7.24%) means unless you state (12.43% yes vs 7.24% no).

**Discussion**

*Pirated academic accounts and use of Sci-Hub section*. To appreciate the similarities and differences between Sci-hub and pirated accounts, please define and explain these two factors in the methods section for the reader. To help the reader understand the significance of the results presented in the first and second paragraphs, please explain how the use Sci-Hub might contrast with access to relevant literature provided by the participating medical schools. In the second paragraph, the fifth sentence begins with ‘This’. Please specify the subject at the beginning of this sentence so the reader can appreciate what might be homogeneous and better understand the point made in the final sentence in the paragraph. In the last paragraph of this section, please make links between the finding about pirated academic accounts in the first sentence and the statements that follow it. Please help the reader to understand how the statements help the reader to understand the significance and implications of this finding.

 The discussion in the sections about c *ourses in writing, English proficiency, and affiliated to a scientific medical student society* do a good job of relating the findings to the local context and the literature. Please consider how the discussion can be broadened to address how your findings might be used by others to generate insights into their own local context or make suggestions about how the findings might offer insights to educators and other educational researchers about the factors from medical training associated with producing a scientific publication during undergraduate study. Since this is a medical and health professions education journal, our readers are interested in the implications for students, teachers, and medical programme curriculum and resourcing.

*Limitations section*. Please give an example to explain to the reader what an undifferentiated classification of the outcome and an increase in residual confusion. Please remember the readers are clinical educators who are not necessarily biomedical scientists or statisticians. Please explain the possible influence of FELSOCEM on the results and how it may bias the results. What factors might be missing in these results that might give readers further insights into the problem of producing scientific publications during medical training. Please outline a few future directions that you or other researchers might take with the findings or the study design to extend our understanding of this problem and topic

**Conclusion**

 Please revise the very long final sentence into at least three shorter ones to help the reader appreciate the important concluding points.

Is the work clearly and accurately presented and does it cite the current literature?

Partly

If applicable, is the statistical analysis and its interpretation appropriate?

Partly

Are all the source data underlying the results available to ensure full reproducibility?

Yes

Is the study design appropriate and is the work technically sound?

Yes

Are the conclusions drawn adequately supported by the results?

Yes

Are sufficient details of methods and analysis provided to allow replication by others?

Yes

Reviewer Expertise:

My research area encompasses teaching, learning, curriculum, and faculty development in health professions education.

I confirm that I have read this submission and believe that I have an appropriate level of expertise to confirm that it is of an acceptable scientific standard, however I have significant reservations, as outlined above.

# Response

**Published:** 2022-05-03

**Authors:** Annel Rojas Alvarado

**Affiliations:** Emerge, Emerging Diseases and Climate Change Research Center, Peru

" **Abstract**

 The authors succinctly outline the topic, a problem that is addressed by the methods, and present the main results. Missing from the abstract is a few sentences outlining the discussion section. Please consider outlining the key discussion points related to the significance and implications of the results for clinical educators who will be reading this article."

**Response: **Thank you. A sentence on the significance and implications of the results has been added to the end of the conclusion section.

 " **Introduction**

 Second paragraph, awkward word choice: I'm not certain what 'stands' means in this sentence. Please consider revising this long sentence into two shorter ones. The second sentence could begin: "SOCIMEP holds international,...""

**Response: **Thank you. The overall sentence was revised.

 "The introduction provides a well-argued warrant for the study by established the local need for the study. As a reader from outside Latin America, I am wondering how this situation might be similar and different to other regions. Please consider relating this problem to the locations of readers beyond Latin America."

**Response: **Thank you. A sentence about the problem related to other contexts was added in the last paragraph (second sentence).

 " **Methods**

 The aim is clearly stated at the beginning of this section and the methods presented are appropriate to address it."

**Response: **Thank you

*"* ***Population and sampling section.*** Please explain the educational outcome-related evidence that was used to determine the power calculation, or if not, state the reason for why you considered a sample size calculation with an 80% of power and 5% of significance. Please describe the censustype sampling procedure or support the terminology with a reference that describes it to the reader."

**Response: **Thank you. The reason for the sample size calculation with the referred parameters were explained. Census-type sampling was revised to a simpler phrase.

*"* ***Operational procedures section.*** First paragraph, first sentence: Please consider replacing XXX with number or word because it looks like information has been omitted to the reader, or please further explain this reference. Please specify what the ICT project was awarded or to whom the project was awarded. Please consider revising the second to last sentence of the second paragraph to explain how participants completed the surveys to provide responses selected or written by themselves and participants took about 15 minutes to complete it."

**Response: **Thank you. "XXX" was revised to 30 ^th^. The ICTs project was awarded an amount of money for publication (details in first & second sentence).  The second to last sentence of the second paragraph was revised (please refer to this part).

*"* ***Measures section.*** Please clarify the term, ‘self-administered survey’ by providing more information or revising the sentence so it better matches the description of the survey in the operational procedures section."

**Response: **Thank you. The term “self-administered survey” was revised

*"* ***Data analysis section.*** The analyses performed by the authors looks appropriate, however, the explanations are dense and may not be easily understood by readers of this journal. Please revise this section so that it is suitable for clinical educators who are not necessarily biomedical scientists or statisticians. Please clarify in the text below on what statistical basis the covariates were selected so the reader does not need to make inferences about the procedures. Please explain if the forward addition assessed using a LRT and if so, explain if a chi-squared distribution or something else was used. Please also describe the criterion used to include or exclude the covariate. Otherwise, the reader has to make the assumption that this information is encompassed in the ‘all hypotheses…’ statement. Please consider stating the following information more thoroughly and with descriptions meaningful to a clinical educator reader: “We estimated nested models following a manual forward selection method to identify covariates associated with self-reported manuscript publication until reaching a parsimonious multivariable model. These covariates were selected using likelihood ratio tests. Crude and adjusted prevalence ratios (PR) were estimated with 95% confidence intervals (CI 95%). All hypotheses were contrasted using 5% significance”"

**Response: **Thank you. We clarified the statistical basis for the forward selection method and other procedure details so that the information is meaningful to clinical educator readers.

*"* **Results**

 First paragraph, first sentence: Please resolve the following contradiction: The methods state that participants completed surveys, however, the first sentence of the results states that interviews were conducted. Both cannot be true."

**Response: **Thank you. The term “were interviewed” was clarified to “completed the survey” to avoid the contradiction.

 "Please consider revising the first, second, third, and fourth paragraphs of the results section so the information presented in them is not repeated in the tables of results as well. Instead, please draw the reader’s attention to important or relevant proportions, relationships, differences, and similarities among the results. In the third paragraph, the percentage stated for the prevalence of scientific publications among first-year and last-year medical students (13% vs 4.3%, respectively) is backwards from the Table 2. The remaining differences need to have the two categories identified. For example, the reader does not know by reading the sentence what being affiliated to a Scientific Medical Student Society (12.43% vs 7.24%) means unless you state (12.43% yes vs 7.24% no)."

**Response: **Thank you. Relevant results were highlighted in the text. In addition, the results in Table 2 were revised and categories were included with their corresponding percentages.

**Discussion**

*"* ***Pirated academic accounts and use of Sci-Hub section.*** To appreciate the similarities and differences between Sci-hub and pirated accounts, please define and explain these two factors in the methods section for the reader. To help the reader understand the significance of the results presented in the first and second paragraphs, please explain how the use Sci-Hub might contrast with access to relevant literature provided by the participating medical schools. In the second paragraph, the fifth sentence begins with ‘This’. Please specify the subject at the beginning of this sentence so the reader can appreciate what might be homogeneous and better understand the point made in the final sentence in the paragraph. In the last paragraph of this section, please make links between the finding about pirated academic accounts in the first sentence and the statements that follow it. Please help the reader to understand how the statements help the reader to understand the significance and implications of this finding."

**Response: **Thank you. Use of Sci-Hub and use of pirated accounts were defined and explained in the methods section. The contrast between Sci-Hub usage and access provided by medical schools is detailed in paragraph 1 of the referred section. The subject was specified in the fifth sentence of the second paragraph. Sentences in the last paragraph have been revised to better link them to the first sentence of this part.

 "The discussion in the sections about courses in writing, English proficiency, and affiliated to a scientific medical student society do a good job of relating the findings to the local context and the literature. Please consider how the discussion can be broadened to address how your findings might be used by others to generate insights into their own local context or make suggestions about how the findings might offer insights to educators and other educational researchers about the factors from medical training associated with producing a scientific publication during undergraduate study. Since this is a medical and health professions education journal, our readers are interested in the implications for students, teachers, and medical programme curriculum and resourcing."

**Response: **Thank you. Suggestions were added at the end of each paragraph in the referred sections.

*"* ***Limitations section.*** Please give an example to explain to the reader what an undifferentiated classification of the outcome and an increase in residual confusion. Please remember the readers are clinical educators who are not necessarily biomedical scientists or statisticians. Please explain the possible influence of FELSOCEM on the results and how it may bias the results. What factors might be missing in these results that might give readers further insights into the problem of producing scientific publications during medical training. Please outline a few future directions that you or other researchers might take with the findings or the study design to extend our understanding of this problem and topic"

**Response: **Thank you. Undifferentiated classification of the outcome and residual confounding were detailed. The influence of FELSOCEM was also explained. A brief list of relevant factors were added. Future directions were outlined.

 " **Conclusion**

 Please revise the very long final sentence into at least three shorter ones to help the reader appreciate the important concluding points."

**Response: **Thank you. The final sentence was revised to three shorter ones.

# Peer review: Reviewer response for version 1

**DOI:** 10.5256/f1000research.29360.r75348

**Published:** 2020-12-14

**Authors:** Virgilio Efrain Failoc Rojas

**Affiliations:** 1 Research Unit for the Generation and Synthesis of Evidence in Health, Saint Ignatius of Loyola University, Lima, Peru

This study brings a broad view of the current efforts of local Scientific Societies of Medical Students on promoting research among medical students. The authors found relevant factors associated with producing a scientific manuscript during medical school including the use of pirated academic accounts, training in basic research skills, English proficiency, and ultimately being affiliated to a scientific medical student society.

 All of these conditions are validated by the faculties of medical schools across Latin America, and are linked between them. The outreach of this study is to give us a sight of factors to continue promoting research capacities among medical students and take advantage of the Medical Students Scientific Society as an ally in this mission.

Is the work clearly and accurately presented and does it cite the current literature?

Yes

If applicable, is the statistical analysis and its interpretation appropriate?

Yes

Are all the source data underlying the results available to ensure full reproducibility?

Yes

Is the study design appropriate and is the work technically sound?

Yes

Are the conclusions drawn adequately supported by the results?

Yes

Are sufficient details of methods and analysis provided to allow replication by others?

Yes

Reviewer Expertise:

Infectious diseases, Health interventions, Systematic Reviews and meta-analysis, Medical education.

I confirm that I have read this submission and believe that I have an appropriate level of expertise to confirm that it is of an acceptable scientific standard.
